# The Etiology of the Thrombotic Phenomena Involved in the Process of Coronary Artery Disease—What Is the Role of Thrombophilic Genes in the Development of This Pathology?

**DOI:** 10.3390/ijms25105228

**Published:** 2024-05-11

**Authors:** Iris Bararu Bojan, Stefan Dobreanu, Maria Cristina Vladeanu, Manuela Ciocoiu, Codruta Badescu, Carmen Plesoianu, Nina Filip, Dan Iliescu, Otilia Frasinariu, Andrei Bojan, Razvan Tudor, Oana Viola Badulescu

**Affiliations:** 1 Department of Pathophysiology, University of Medicine and Pharmacy Gr. T. Popa, 700115 Iași, Romania; iris_bararu@yahoo.com (I.B.B.); zamosteanu_nina@yahoo.com (N.F.); violabadulescu@yahoo.com (O.V.B.); 2 Recuperare Hospital Iasi, University of Medicine and Pharmacy Gr. T. Popa, 700115 Iași, Romaniaminerva.badescu@umfiasi.ro (C.B.); 3 Department of Internal Medicine, University of Medicine and Pharmacy Gr. T. Popa, 700115 Iași, Romania; 4 Department of Pediatry, University of Medicine and Pharmacy Gr. T. Popa, 700115 Iași, Romania; 5 Department of Surgical Sciences, University of Medicine and Pharmacy Gr. T. Popa, 700115 Iași, Romania; 6 Department of Orthopedy, University of Medicine and Pharmacy Gr. T. Popa, 700115 Iași, Romania

**Keywords:** thrombophilia, coronary artery disease, arterial thrombosis, factor V Leiden, prothrombin mutation, plasminogen activator inhibitor 1, antiphospholipid syndrome, hyperhomocysteinemia

## Abstract

Cardiovascular diseases, among which includes coronary artery disease, represent one of the most important causes of mortality and morbidity worldwide. Research aimed at determining the risk factors involved recognizes a group of “traditional” risk factors, but also more recent studies identified over 100 “novel” ones which may have a role in the disease. Among the latter is the thrombophilia profile of a patient, a pathology well-established for its involvement in venous thromboembolism, but with less studied implications in arterial thrombosis. This paper reviews the literature, explaining the pathophysiology of the thrombophilia causes associated most with coronary thrombosis events. Results of several studies on the subject, including a meta-analysis with over 60,000 subjects, determined the significant involvement of factor V Leiden, prothrombin G20210A mutation, plasminogen activator inhibitor–1 and antiphospholipid syndrome in the development of coronary artery disease. The mechanisms involved are currently at different stages of research, with some already established and used as therapeutic targets.

## 1. Introduction

Cardiovascular diseases (CVD) are the leading global cause of mortality, with thrombotic complications playing a major role [1,2]. One of the most incriminated mechanisms of CVD is the atherosclerotic process, in which lipids accumulate at the subendothelial level, resulting in a low-grade inflammatory lesion and transforming the endothelium into a phenotype prone to inflammation and thrombosis [2]. One of the most common forms of CVD that leads to an important burden on the health system is coronary artery disease.

Coronary artery disease (CAD) is itself the leading cause of morbidity and mortality in both developed and developing countries and can be attributed to a number of causes and risk factors [2,3]. Since the identification of the main “traditional” risk factors for CAD by the Framingham study, more than 100 “novel” risk factors have been proposed and studied as potential causative agents, triggers or therapeutic targets. Clotting markers represent some of these “novel” markers that have generated significant interest through their association with acute coronary syndromes (ACS) in young patients [4,5,6,7].

This article aims to identify the most common markers of hypercoagulability associated with CAD and to provide an insight into the pathophysiology of the disease.

The Framingham Study, one of the largest prospective studies, which began enrolling participants in 1948, provided the classic view of CAD used today by identifying the main predisposing factors, their incidence and prevalence, and their role in CVD pathology [4,5]. These have come to be known as “traditional” risk factors and have since been the main therapeutic target in the prevention of CVD. Several newly identified risk factors are classified as “novel” and represent more or less studied risk markers that may impact overall CVD risk [4,5,6]. Both traditional and new risk factors are listed in Table 1.

Many risk factors for cardiovascular disease have been identified, of which some can be modified and some cannot. CVD can be defined as a set of distinct diseases whose underlying cause of development is most often atherosclerosis, therefore the risk factors are similar. Among the modifiable risk factors identified are elevated blood pressure, high blood cholesterol levels, diabetes control, smoking, obesity, sedentary lifestyle. But others, such as age, sex (male predisposition), family history or ethnicity cannot be influenced in any way [8,9,10].

Risk factors identified in a cohort of young survivors of acute coronary syndromes (ACS) (men < 45 years and women < 50 years) create a profile of a patient who is more likely to be male, a smoker, dyslipidemic, with a positive family history of VD, while less likely to be hypertensive or diabetic. Angiographic data collected from these patients indicate that the incidence of coronary atherosclerosis is lower than in older ACS patients [11].

Traditional risk factors alone cannot explain why different populations have been affected differently by CAD, so non-traditional risk factors have developed a high level of interest. Of these, particular attention has been paid to a group of hematological risk factors due to their induction of a hypercoagulable state, translated into thrombus formation and predisposition to CAD [4,6,12,13].

Thrombophilia is a hypercoagulable state usually associated with venous thrombosis; however, the pathophysiology and risk factors of CAD are shared with deep vein thrombosis and pulmonary embolism.

In patients who develop unprovoked arterial thrombosis at an early age, evaluating for thrombophilia may be necessary, as certain conditions have been associated with coronary, cerebral and peripheral ischemia [14,15,16,17,18]. Arterial system thromboses have been associated with a number of hematological conditions, such as factor V Leiden (FVL) mutation, prothrombin G20210A mutation or methylenetetrahydrofolate reductase (*MTHFR* C677T) mutations. Among other conditions, arterial thrombosis has also been associated with reduced protein C activity, antiphospholipid syndrome (APS), hyperhomocysteinemia or elevated factor VII [18,19,20,21,22,23,24].

Thrombophilia is a prevalent problem, with studies identifying up to 10% of the population as carrying a form of hereditary thrombophilia when there is a positive family history [25]. Another more recent study by Khider et al. provided clues to the prevalence of hereditary thrombophilia in the general population. The results are shown in Table 2 [26].

A retrospective cohort study by Omran et al. evaluating hypercoagulability in patients with ischemic stroke found that 43% of enrolled patients tested positive for at least one test. The most common abnormality observed was heterozygosity of the *MTHFR* gene mutation, lupus anticoagulant identification and antithrombin deficiency [27].

Therefore, thrombotic complications play a significant role in CVD, particularly in CAD, which stands as the foremost cause of global mortality. While traditional risk factors such as age and hypertension have long been acknowledged, newer markers of hypercoagulability are garnering attention for their association with acute coronary syndromes (ACS). Thrombophilia, typically linked with venous thrombosis, also poses a risk for arterial thrombosis, including CAD. Evaluation for thrombophilia becomes crucial in instances of early-onset arterial thrombosis, where factors like FVL mutation or hyperhomocysteinemia may be implicated. Studies suggest a notable prevalence of hereditary thrombophilia, highlighting its importance in CAD pathology and urging further investigation into preventive and therapeutic measures.

Our narrative review centers on key markers of hypercoagulability implicated in CAD. Thus, we delve into the pathophysiology of thrombophilia, emphasizing factors contributing to heightened clotting and stressing the significance of genetic predisposition. Additionally, we aim to elucidate the current relevance of thrombophilia testing in CAD while offering insights into future perspectives on this matter.

## 2. Literature Search

We have realized a narrative review regarding the importance of the thrombophilic status in the pathogeny of CAD.

A systematic search of all published studies related to thrombophilia and CAD was performed in PubMed to identify the status of research on the different mechanisms of thrombophilia involved in CAD, using the following keywords: arterial thrombosis, thrombophilia, coronary artery disease.

Accordingly, another PubMed search was performed to obtain significant articles on the pathophysiological mechanisms of hypercoagulability causes previously identified in CAD. The following keywords were used: factor V Leiden, prothrombin mutation, plasminogen activator inhibitor-1 (PAI-1), antiphospholipid syndrome, hyperhomocysteinemia, antiphospholipid syndrome.

Studies were excluded if they were not written in English, did not involve human subjects, included single case reports.

Screening of search results was divided into three steps: review of titles and abstracts, assessment of full manuscripts for eligibility, and inclusion of studies for this review.

## 3. Results and Discussion

### 3.1. Pathophysiology

Thrombin is generated on the surface of activated platelets as a result of the coagulation cascade activated by subendothelial tissue factor (TF). The interaction between TF and factor VII forms an active complex which in turn activates factors IX and X. Activated factor X causes proteolytic formation of thrombin from its precursor prothrombin, which will lead to fibrin formation. The initiation of the coagulation cascade is regulated by tissue factor pathway inhibitor (TFPI), which acts on both TF-factor VII complex and activated factor X [28,29].

**Figure 1:**
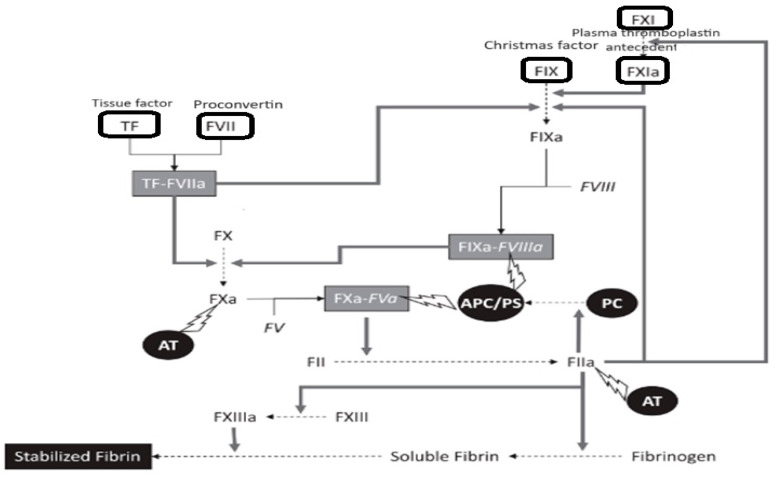
Scheme of the coagulation system; TF: tissue factor; AT: antithrombin; APC: activated protein C; PS: protein S, PC: protein C, adapted from [31].

TF is a protein that is found in abundance in atherosclerotic plaques, thus favoring the establishment of conditions for a hypercoagulable state. At the same time, overexpression of TFPI has been shown to be beneficial in reducing thrombus formation following vascular injury in animal models [2,30]—Figure 1.

The involvement of thrombin in CAD can be explained by its role in impairing endothelial barrier function. Thrombin promotes endothelial cell contraction, which leads to increased permeability, leading to plasma extravasation and oedema. Other studies have suggested that thrombin influences the production of reactive oxygen species, thereby promoting atheromatous plaque formation [32,33,34].

Thrombin may also lead to increased levels of monocyte chemoattractant protein, a chemokine found in higher amounts in atherosclerotic plaques. Thrombin is also known to exert proatherogenic properties by activating cells and mechanisms such as protease-activated receptor 1, cytokine and chemokine production, vascular calcification, increasing endothelial permeability and promoting vascular smooth muscle cell migration and proliferation, platelet activation or leukocyte recruitment and adhesion [32,34,35,36]. Furthermore, Borissoff et al. conducted a study showing that control of the clotting process by thrombin inhibition may be a future therapeutic target in atherosclerosis therapy [37].

Ye et al. coordinated one of the largest meta-analyses on this topic in terms of study population; it included data from 191 studies, reaching a total of more than 150,000 patients (cases + controls), thus providing insight into the influence of thrombophilia markers in CAD patients.

The study focused on the analysis of seven hemostatic gene polymorphisms:

- the G→A substitution at position 1691 of the factor V gene, resulting in an arginine to glutamine exchange in codon 506 (commonly referred to as Arg506Gln, factor V Leiden, or R506Q);
- the G→A exchange at position 10,976 in the factor VII gene, which results in an arginine to glutamine exchange in codon 353 (also known as Arg353Gln or R353Q);
- the G→A exchange at position 20,210 in the 3′ untranslated region of the prothrombin gene;
- the 4G/5G insertion/deletion in the PAI-1 gene at a position –675 of the promoter region;
- the C→T substitution at position 807 in the GPIa gene;
- a C→T substitution at position 1565 in exon 2 of the GPIIIa gene, which results in a leucine to proline exchange in codon 33 (Leu33Pro or PlA1/A2);
- the T→C substitution recently identified at position –5 upstream of the ATG initiation codon in the GPIb gene (the von Willebrand factor-binding subunit of the complex) [38].

Following analysis of the results, strong, weak and null associations of each marker with CAD can be observed. The most significant association is that of two genes: the factor V gene and the 20210A variant of the prothrombin gene, both leading to an increase in circulating thrombin. Also, a positive but weak association may also consist in the 4G variant of the PAI-1 gene [38]. There is no evidence to support a relationship between CAD risk and the 10976A variant of the factor VII gene, as well as with three polymorphisms of the platelet receptor genes GPIa, Ib and IIIa [38].

#### 3.1.1. Factor V Leiden

**Figure 2:**
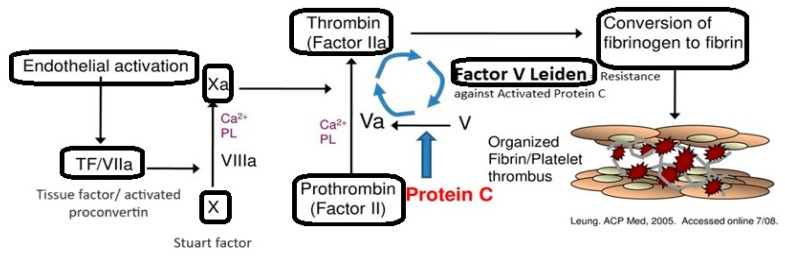
Factor V Leiden—mechanism of action; TF: tissue factor; PL: phospholipids—adapted from Leung. ACP Med. 2005 [45].

Factor V Leiden is the mutant form of factor V that is encoded by a gene located on chromosome 1 (1 q21-25). It is transmitted in an autosomal dominant manner with incomplete penetrance, which means that not all carriers of the mutation will develop the disease. Single nucleotide polymorphisms of the factor V gene, leading to an R506Q mutation, are the most common inherited abnormality [32,39,40,41]. Physiologically, factor V is synthesized in the liver and activated by thrombin, leading to the conversion of prothrombin to thrombin [39,42]. Activated protein C uses protein S as a cofactor to cleave and degrade activated factors V and VIII. APC resistance represents the inability of protein C to inhibit these factors, thus allowing longer thrombin generation times. The most common cause of APC resistance is the FVL mutation, which alters the binding site of APC, rendering it inactive, thus leading to a hypercoagulable state [41,43,44]—Figure 2.

In patients carrying multiple thrombophilic defects, the risk of complications is synergistically increased. Studies have shown that the association between VFL and 20210A defect increases the relative risk of venous thromboembolism by almost 5-fold compared to each risk alone [45,46].

#### 3.1.2. Prothrombin G20210A Mutation

Prothrombin thrombophilia is a condition caused by a point mutation in the prothrombin gene and is transmitted in an autosomal dominant manner, with homozygous variants having a higher risk of thrombotic complications than heterozygous variants [47,48]. DNA sequencing performed on the prothrombin gene in patients with venous thromboembolism (VTE) found a single guanine to adenine mutation at nucleotide position 20,210 in the 3′ region of the prothrombin gene. The mutation does not affect the structure or function of the prothrombin molecule, nor its conversion to thrombin, but produces elevated levels of thrombin [47,48].

The G20210A mutation of prothrombin results in an increase in mRNA, hence increased protein expression, which in turn leads to overproduction of thrombin-activated fibrinolysis inhibitor (TAFI), an inhibitor of the fibrinolysis process [48,49,50]. Increased levels of prothrombin in the blood tend to promote clotting, which often has the clinical expression of VTE. Previous studies have shown that an increase of more than 115% in normal prothrombin levels leads to a 2-fold increased risk of VTE [48,49,50]. Although venous complication has been studied in more detail, the latest trend is to investigate the role played in episodes of arterial thrombosis. However, several investigators have reported a significant increase in the prevalence of the G20210A variant of the prothrombin gene in CAD patients (1.8% to 12.5%) compared with newborns or age-matched controls and a 4-fold increase in the risk of myocardial infarction in young women with this variant [38]. Overall, a significantly elevated risk of CAD was associated with the prothrombin G20210A polymorphism, as demonstrated by a meta-analysis by Jin et al. including 39 eligible cases [51].

#### 3.1.3. Plasminogen Activator Inhibitor-1 (PAI-1)

PAI-1 is a serine protease inhibitor, also known as serpin E1, encoded by the SERPINE1 gene, located on chromosome 7 (7q21.3-q22). It functions as the main inhibitor of tissue plasminogen activator, a component of the fibrinolytic system. PAI-1 deficiencies have been shown to accelerate the rate of fibrinolysis and bleeding, while increased levels of PAI-1 predispose to vascular thrombosis [52,53]. In vivo, the highest concentrations of PAI-1 are found in platelet granules, where nearly 90% of circulating PAI-1 is found. Other sources may be endothelial cells, macrophages or adipocytes, all of which can contribute to PAI-1 levels in humans. Their regulation can be affected by various pathological conditions such as vascular disease, inflammation or sepsis [53].

As a major inhibitor of fibrinolysis, PAI-1 may play a role in promoting atherosclerosis and thrombosis, leading to pathologies such as cerebrovascular disease or CAD, an assumption confirmed by its strong expression in atherosclerotic lesions. High concentrations of PAI-1 predispose to the development of atherosclerotic plaques with a high ratio of lipids to vascular smooth muscle cells, making them prone to rupture and subsequent complications [52,53,54,55]. In addition, a significant association has been observed between elevated PAI-1 levels and conditions known to favor a prothrombotic state, such as hypertension, diabetes or obesity [52]. A study on predictors of myocardial infarction identified elevated PAI-1 levels in these patients. However, after adjusting the results to account for markers of metabolic syndrome, such as BMI, triglyceride levels and LDL-C, the predictive value of PAI-1 disappears, suggesting that PAI-1 may actually be a marker of metabolic syndrome, which in turn influences CAD risk [38].

#### 3.1.4. Hyperhomocystinemia

Homocysteine is α-nonproteogenic amino acid, biosynthesized from methionine by two major metabolic pathways: remethylation and transsulfuration, in the presence of folate and vitamin B_12_. The first pathway, remethylation, uses methylenetetrahydrofolate reductase (MTHFR) to convert homocysteine to methionine [56,57]. A C677T point mutation in the gene encoding *MTHFR* is the most common genetic cause of hyperhomocysteinemia. Homozygous carriers may have a moderate increase in homocysteine levels and may experience varying degrees of symptoms caused by venous or arterial thrombosis [57,58]. Symptoms can also be attributed to a lack of vitamins B_6_, B_12_ or folate. These components are pivotal in the homocysteine pathway, and their absence would hinder homocysteine processing, resulting in its buildup [58,59].

High homocysteine levels have toxic effects on the vascular endothelium, damaging it and causing cellular dysfunction, followed by platelet activation, thrombus formation, thus creating a hypercoagulable state. The precise mechanism by which the endothelium is affected is still partly unknown and more research is needed to better understand it [48,60]. Some studies have shown that the 677T *MTHFR* genotype is linked to elevated serum homocysteine levels, while others have shown a link between hyperhomocysteinemia and arterial thrombosis. However, attempts to directly correlate the *MTHFR* mutation with arterial thrombosis events, thus linking genotype to clinical disease, have failed to establish a significant association. In addition, arterial ischemic events were not reduced by lowering homocysteine levels in these patients [60,61].

#### 3.1.5. Antiphospholipid Syndrome

Antiphospholipid syndrome (APS) is a hypercoagulable condition defined by recurrent thrombotic episodes, including deep vein thrombosis (DVT), pulmonary embolism, placental thrombosis leading to miscarriage or arterial thrombosis, all in the presence of circulating antiphospholipid antibodies (APA). For the diagnosis of APS, the patient must have one of the following clinical presentations: arterial or venous thrombosis, pregnancy loss or thrombocytopenia, associated with at least one of the following laboratory criteria: presence of lupus antibodies, moderate/high titer of IgG/IgM anticardiolipin antibodies or IgG/IgM anti-glycoprotein B2 antibodies; each confirmed on two separate occasions with a minimum interval of 12 weeks [62,63,64].

The hypercoagulable state induced by APS is a consequence of the reaction between negatively charged phospholipid antigens (e.g., anticardiolipin antibody, anti-B2 glycoprotein) and immunoglobulins. These bind to the surface of endothelial cells, causing their activation and increasing the expression of adhesion molecules on their surface. Furthermore, antiphospholipid antibodies that bind to endothelial cells inhibit prostacyclin secretion, leading to increased platelet adhesion and TF release by vascular endothelium and monocytes. As a result, protein C activity is inhibited, favoring further secretion of IAP, leading to inhibition of the fibrinolytic system [62,63,64]. A study by Gurlek et al., which included patients with CAD and APS who were treated by percutaneous coronary intervention (PCI), showed that restenosis rates at 1-year follow-up were significantly higher for those patients who had moderate or high titers of anticardiolipin antibodies. This finding was also confirmed by other studies, which observed a higher rate of post-PCI stent restenosis and a higher target vascular reconstruction rate in patients with both APS and CAD [65].

### 3.2. Thrombophilia Testing

Thrombophilia screening is a comprehensive assessment of the patients’ state and is performed after an unprovoked thromboembolic event, usually VTE. An unprovoked event occurs without an identifiable cause, as opposed to provoked thrombotic events, which may be the result of transient or persistent, minor or major risk factors [66,67,68,69]. The purpose of a screening profile for thrombophilia is to identify a possible cause of thrombosis, to estimate the risk of recurrence, to guide therapeutic measures or as part of primary prophylaxis for family members [66,67].

VTE, being more common than arterial thrombosis, has been studied more extensively. Thus, more data are available for its management. For this reason, most indications for thrombophilia testing are formulated as a consequence of VTE episodes and not arterial thrombosis [69,70]. Current indications for thrombophilia testing are listed below.

- First VTE event < 40 years;
- VTE at unusual sites < 50 years; (e.g., upper limb thrombosis, retinal vein thrombosis);
- First unprovoked event < 60 years;
- Recurrent episodes of VTE;
- Estrogen therapy/pregnancy—sole risk factor;
- Recurrent superficial vein thrombosis without varicose veins;
- Asymptomatic first-degree relatives of symptomatic patients with proved thrombophilia [69,70,71,72].

In terms of markers to be tested, those associated with strong and independent risk factors, which possess known mutations and are quite common in the general population, have been proposed. These include protein C and protein S, antithrombin, FVL or prothrombin gene mutations. Conditions associated with a low prevalence or low thrombosis risk are not included in the screening profile. These include testing for increased activity of factors VII, IX, XI, elevated PAI-1 or *PAI-1* polymorphisms or *MTHFR* mutations; the latter has been identified by one study as present in up to 45% of the general population and is also considered a poor risk factor for thrombosis [69,71,73,74]. Testing for antiphospholipidic syndrome (APS) is also included in most screening profiles for thrombophilia because it is an important acquired risk factor for venous and arterial thrombosis. According to the definition of APS, at least one of its markers must be identified: lupus anticoagulant, IgG/IgM anticardiolipin antibody or IgG/IgM antiglobulin B2 antibody [62,74].

### 3.3. The Dysregulated Coagulopathy Induced by Procoagulant Platelets

Procoagulant platelets play a pivotal role in thrombotic disorders by facilitating the assembly of coagulation factors on their membrane surface. This process is essential for generating the thrombin burst and initiating fibrin formation, thus bridging primary and secondary hemostasis. Platelets, along with plaque characteristics, determine the blood’s prothrombotic potential and influence the degree of intraluminal thrombus formation, ranging from partial to occlusive, especially in CAD patients, who exhibit a significantly heightened response to 5 μm adenosine diphosphate (ADP) compared to healthy controls.

Under the influence of potent agonists, a subset of platelets can transition to a procoagulant state, contributing to coagulation and fibrin formation. The composition of fresh coronary thrombi, consisting of erythrocytes, neutrophils, and aggregated platelets embedded in a fibrin meshwork, underscores the importance of fibrin stabilization in occlusive coronary thrombosis. However, standard platelet assays and antiplatelet therapies predominantly target aggregation and platelet activation rather than procoagulant function. Selectively inhibiting platelet procoagulant activity could offer a more targeted approach to managing coronary thrombosis, specifically by impeding thrombin generation within the thrombus without affecting endothelial fibrin formation. Recent research has demonstrated that the necrosis marker GSAO, combined with the platelet activation marker P-selectin, effectively identifies agonist-induced procoagulant platelets both in vitro using a washed platelet assay and in vivo in mouse thrombosis models. Notably, the cell death marker 4-[N-(S-glutathionylacetyl)amino]phenylarsonous acid GSAO has revealed a positive correlation between procoagulant platelets, platelet thrombus size, and fibrin formation [75].

### 3.4. Future Perspectives Regarding the Thrombophilia Assessment in Coronary Artery Disease

Looking forward, the future of assessing thrombophilia in CAD holds promising advancements. By incorporating innovative biomarkers and imaging technologies, we can gain a more thorough understanding of thrombotic risk and tailor treatments accordingly. For instance, combining advanced imaging methods like intravascular ultrasound (IVUS) and optical coherence tomography (OCT) with biomarkers for platelet activation and procoagulant function could improve our ability to identify plaque vulnerabilities and predict thrombotic events more accurately. Additionally, emerging technologies such as microfluidic assays and point-of-care devices offer the potential for quick and precise detection of platelet dysfunction and hypercoagulability, enabling timely intervention and risk assessment for CAD patients. Moreover, integrating genetic profiling and machine learning algorithms may refine risk-prediction models, pinpointing individuals at high risk who could benefit from more aggressive antithrombotic therapy or targeted interventions. Collaboration among clinicians, researchers, and industry partners will be essential in translating these innovations into clinical practice, ultimately enhancing outcomes for CAD patients by reducing thrombotic complications.

## 4. Conclusions

Since the first identification of environmental risk factors that play a role in the development of coronary heart disease, a wealth of new evidence has provided a clearer picture of this pathology. Researchers have proposed and analyzed new genetic risk factors; in particular, factors involved in the maintenance of physiological hemostatic balance have been proposed and analyzed in an increasing number of studies as having an influence on patient risk of thrombotic complications, including CAD. This evidence is the result of numerous epidemiological studies as well as clinical observations and case reports. However, despite the abundance of data, more studies are needed to assess gene-environment and gene-gene interactions, in particular to determine the causal role of certain risk factors.

Complications from hypercoagulable states are known to be implicated in the majority of cases of acute coronary, cerebrovascular and peripheral arterial disease events. Although atherosclerosis is undoubtedly implicated in most of these events, the mechanisms leading to atherosclerotic plaque formation are still under investigation. The role of hemostatic factors in atherosclerotic lesion formation has been difficult to demonstrate, due to conflicting results and limited data from recent studies. However, some factors have shown a stronger association with CAD, including factor VII, PAI-1, prothrombin mutations, von Willebrand factor or fibrinogen. The role of factor V gene polymorphisms in CAD is controversial, with different genetic variants and genotypes showing different results in the groups analyzed, and some studies even failing to identify a significant association [73,74,75]. The anticoagulant activity of FV, which plays a major role in thrombin regulation, is impaired by these mutations, leading to a malfunction of FV protein and thus increased thrombin concentrations, all while the factor V Leiden mutation has been associated with increased resistance to PC. The hypercoagulable state thus created and the increased thrombin concentrations aid the mechanisms of atherosclerosis development and progression.

The results of a large meta-analysis also indicated a weak, but still positive, association of the PAI-1 gene with CAD [76]. Increased levels of PAI-1 are associated to accelerated atherosclerosis with increased lipid content compared to vascular smooth muscle cells, thus leading to complications such as plaque rupture and thrombotic complications. The aim of these studies is to further expand our understanding of the underlying mechanisms involved in the grand scheme of what CAD is, but also to have an impact on the therapeutic tools available to physicians to offer patients increasingly effective management practices. An example of a possible therapeutic target in patients with identified FVL mutations would be selective targeting of factor V with maintenance of physiological hemostasis.

It is well-known that rupture or erosion of a vulnerable atherosclerotic plaque is followed by thrombosis. The prothrombotic state is the result of the association between endothelial cell damage and hypercoagulability of the blood, which may induce flow alteration due to the geometrical features and location of the plaque. Complications of coronary atherosclerotic plaque must be present for an event to occur, but also the presence of prothrombotic state at the site of plaque rupture or erosion is necessary to cause a thrombotic event. It is important to determine whether thrombophilia testing would be beneficial for the diagnosis and treatment of CAD. For example, the ROADMAP-CAD study is a prospective study that seeks to evaluate the utility of measuring hypercoagulability biomarkers to better diagnose severe coronary artery disease. This research by Gerotziafas et al. showed promising results indicating that hypercoagulability assessment could be proposed to screen patients with severe CAD [76,77,78]—Table 3.

The present findings reinforce the need for larger studies and well-designed meta-analyses to reliably assess sufficient information to eliminate the bias seen in small group studies and case reports. In conclusion, certain thrombophilic phenotypes are associated to varying degrees with CAD. Although our current knowledge and understanding of the pathology has progressed significantly, there is still a need for future studies to focus on the interaction between individual, traditional, genetic and novel cardiovascular risk factors, among which thrombophilic defects play a substantial role.

Thrombophilia emerges as a crucial factor in the complex landscape of CAD, offering valuable insights into its pathogenesis and clinical manifestations. Thrombophilia not only underscores the thrombotic complications inherent in CAD but also sheds light on the mechanisms underlying arterial thrombosis, guiding diagnostic and therapeutic strategies. The prevalence of hereditary thrombophilia further emphasizes its significance in CAD pathology, suggesting the need for comprehensive evaluation and tailored interventions to mitigate thrombotic risk and improve clinical outcomes in individuals predisposed to this condition. Ultimately, the recognition and management of thrombophilia represent a crucial aspect of CAD care, offering opportunities for targeted prevention and treatment strategies to mitigate the burden of this leading cause of global morbidity and mortality.

## Figures and Tables

**Table 1:** Cardiovascular risk factors [4].

Cardiovascular Risk Factors	
**Traditional Risk Factors**	**Non-Traditional Risk Factors**
Age: > 45 years (M)/> 55 years (F)	Left ventricular hypertrophy
Family history of CVD	Hyperhomocysteinemia
Smoking	Lipoprotein(a) excess
Hypertension (BP > 140/90 mmHg)	Hypertriglyceridemia
LDL-C (130–159 mg/dL) + at least 2 risk factors	Oxidative stress
HDL-C (< 35 mg/dL)	Hyperfibrinogenaemia
Obesity (BMI > 30 kg/m^2^)	Procoagulant substances (e.g., Plasminogen, PAI-1, vWF, factor VII)
Diabetes mellitus	Infectious agents (e.g., *Chlamydia pneumoniae*, *Helicobacter pylori*, cytomegalovirus)

**Table 2:** Prevalence of inherited thrombophilia [26].

Inherited Thrombophilia: Prevalence in the General Population
Severe Thrombophilia:
Antithrombin deficiency: 0.02–0.2% FVL (homozygous): 0.02% Double heterozygous (FVL and F2 c.*97G>A): Less than 0.1% Protein C deficiency: 0.2–0.4% Protein S deficiency: 0.03–0.5%
Mild Thrombophilia:
FVL (heterozygous): 3.0–7.0% F2 c.*97G>A (heterozygous): 0.7–4.0%

**Table 3:** Studies discussing the perspective of thrombophilia assessment in CAD.

Reference	Studies	Perspective on Thrombophilia Assessment in CAD
[18]	Milgrom A. et al. (2018). Thrombophilia in 153 Patients with Premature Cardiovascular Disease ≤ Age 45. Clin Appl Thromb Hemost.	high factor VIII
		homocysteine
		ACLA IgM,
		low free protein S
		high Lp(a)
		lupus anticoagulant
[24]	Peng X. et al. (2020). Association of MTHFR Variant C677T and Risk of Carotid Atherosclerosis. BMC Cardiovasc Disord.	*MTHFR* genotypes
[25]	Vagdatli E. et al. (2013). Prevalence of Thrombophilia in Asymptomatic Individuals with a Family History of Thrombosis. Hippokratia.	the activity of ATIII
		protein C and protein S
		FVIII
		FΧΙΙ
		total homocysteine levels
[27]	Omran SS. et al. (2019). Clinical Impact of Thrombophilia Screening in Young Adults with Ischemic Stroke. J Stroke Cerebrovasc Dis.	the anticardiolipin and anti-beta-2 glycoprotein-1 antibodies (IgG and IgM isotypes) and lupus anticoagulant;
		Factor V Leiden gene mutation;
		prothrombin gene G202010A mutation;
		protein C and protein S
		antithrombin deficiencies
		increased homocysteine
		*MTHFR* gene mutation
[38]	Ye Z. et al. (2006). Seven Haemostatic Gene Polymorphisms in Coronary Disease: Meta-analysis. Lancet.	factor V G1691A (i.e., factor V Leiden),
		factor VII G10976A
		prothrombin G20210A,
		plasminogen activator inhibitor-1 (PAI-1) [-675] 4G/5G
		three platelet glycoprotein (GP) receptor variants (GPIa C807T, GPIbalpha T[-5]C, GPIIIa C1565T),
[40]	Klarin D. et al. (2019). Genome-wide Association Analysis of Venous Thromboembolism Identifies New Risk Loci. Nat Genet.	discovery genome-wide association study with testing of approximately 13 million DNA sequence variants
[51]	Jin B. et al. (2011). Association of Prothrombin G20210A Polymorphism with Coronary Artery Disease. Mol Biol Rep.	prothrombin *G20210A* polymorphism
[65]	Gürlek A. et al. (2005). Association between Anticardiolipin Antibodies and Recurrent Cardiac Events in ACS. Int Heart J.	anticardiolipin (aCL) antibodies (Ig M and Ig G)
[76]	Gerotziafas GT. et al. (2020). Prospective Assessment of Biomarkers for Identification of Patients with Severe CAD. Clin Appl Thromb Hemost.	biomarkers of hypercoagulability
